# Sublethal Effects of *Solanum nigrum* Fruit Extract and Its Pure Glycoalkaloids on the Physiology of *Tenebrio molitor* (Mealworm)

**DOI:** 10.3390/toxins10120504

**Published:** 2018-12-01

**Authors:** Marta Spochacz, Szymon Chowański, Monika Szymczak, Filomena Lelario, Sabino A. Bufo, Zbigniew Adamski

**Affiliations:** 1 Department of Animal Physiology and Development, Institute of Experimental Biology, Faculty of Biology, Adam Mickiewicz University in Poznań, ul. Umultowska 89, 61-614 Poznań, Poland; szyymon@amu.edu.pl (S.C.); monikasz@amu.edu.pl (M.S.); ed@amu.edu.pl (Z.A.); 2 Department of Sciences, University of Basilicata, Via dell’Ateneo Lucano 10, 85100 Potenza, Italy; filomenalelario@hotmail.com; 3 Electron and Confocal Microscope Laboratory, Faculty of Biology, Adam Mickiewicz University in Poznań, ul. Umultowska 89, 61-614 Poznań, Poland

**Keywords:** *Solanum nigrum* extract, *Tenebrio molitor*, ultrastructure, midgut, fat body, biochemistry, contractility, heart, oviduct, glycogen, lipids

## Abstract

Background: *Solanaceae* plants produce glycoalkaloids (GAs) that affect various physiological processes of herbivorous insects and they are being tested as potential alternatives for synthetic pesticides. They cause lethal and sublethal effects. Nevertheless, their mode of action remains unclear. Therefore, we examined the effects of *Solanum nigrum* fruit extracts and pure glycoalkaloids on a model beetle, *Tenebrio molitor*. Methods: Plant extracts or pure alkaloids were added to the food of the larvae for three days. The lipid, glycogen, and protein content in the fat body and the midgut were determined, and the contractility of the heart, hindgut, and oviduct muscles was tested using the video-microscopy technique. Finally, the ultrastructure of the fat body and the midgut was observed using electron microscopy. Results: No lethal effects were noted. Sublethal changes were observed in the content of biomolecules, malformations of organelles, chromatin condensation, and heart and oviduct contractility. The observed effects differed between the tested glycoalkaloids and the extract. Conclusions: Both the extract and pure GAs have a wide range of effects that may result in impaired development, food intake, and reproduction. Some early effects may be used as bioindicators of stress. The effects of the extract and pure alkaloids suggest that the substances produced by the plant may act additively or synergistically.

## 1. Introduction

In recent years, the knowledge about the potential alternatives for synthetic pesticides, such as plant derivatives, has significantly increased [1,2,3]. Natural products are already in use on the markets worldwide, for example, in organic agriculture [4]. Some of the active compounds from extracts have been changed structurally to obtain more persistent substances, such as the neem tree (*Azadirachta indica*) extract, which has progressively become increasingly popular [5], or pyrethrins obtained from *Chrysanthemum cinerariifolium*, which in the 1970s became a source for the third class of synthetic pyrethroids [6]. One of the most difficult aspects of crop protection is the application of substances to stored commodities. This step requires easily degradable compounds that are relatively nontoxic to mammals. From an economic point of view, substances that are used in crop protection should be inexpensive and their emission should not lead to their environmental accumulation or to toxic effects on nontarget organisms. Natural substances that are produced by plants to deter herbivores meet the criteria outlined above. They not only are effective in the control of pest insect populations, causing up to 100% mortality at concentrations as low as 0.5 mg/cm^3^ after 24 h of fumigation [7], but also exhibit selective action against various species [8,9]. The effectiveness of plant derivatives may strongly depend on the dosage, method of extraction and solvent [10,11,12], as well as the application method [13]. Given that there are an enormous number of possible plant extracts containing active ingredients and that there are many solvents that can be used, there is a great need to study the possible combinations to obtain the most effective substances for specific species. The majority of research has focused on direct toxic effects, which can be used to limit pest populations [1]. However, understanding the mode of action of plant derived substances can be useful in planning strategies for pest control. The nature of the effects on both the target and nontarget tissues can be tested by exposure of the target species to low doses of the plant-derived substances. Although the effects may be subtle and even statistically non-significant, some discrete malformations and malfunctions may be observed before the massive toxic effects appear when low concentrations of these substances are used [14,15,16]. Furthermore, the sublethal doses and concentrations can reveal the first effects and the mode of action at the level of organs, tissues, or even cells.

In this study, we tested the extract that was obtained from the unripe fruits of *Solanum nigrum*, a plant commonly distributed in Europe, which is known to produce glycoalkaloids (GAs). Previous studies have demonstrated that the extract caused larvicidal effects in mosquitoes (Diptera), such as *Culex vishnui* [17], *Culex quinquefasciatus* [18], *Culex pipiens*, *Aedes caspius* [19], *Anopheles culicifacies*, *Aedes aegypti* [20], and *Anopheles stephensi* [21]. Toxic effects have also been found in the fruitfly (*Drosophila melanogaster*: Diptera) [22] and the Colorado potato beetle (*Leptinotarsa decemlineata*: Coleoptera) [23]. *S. nigrum* extract, in addition to its toxic effects, was reported to have promising anticancer [24] and antimicrobial properties [25]. The tested extract contains 10 GAs, but two, solasonine and solamargine, are present in greatest amounts [22]. Studies have shown not only the toxic influence of glycoalkaloids on animal health [26], but also the beneficial effects, such as anticancer properties [27,28]. Since alkaloids have been reported as promising tools for pest management (for review see: [1,2]), we decided to examine the extract from *S. nigrum* fruits as well as pure solasonine and solamargine and to compare their effects on a model organism in ecotoxicological studies and a pest of stored products—the yellow mealworm beetle *T. molitor*.

We addressed the following research questions:

- Do the extract and the pure GAs cause lethal toxic effects and disturb the development of *T. molitor* larvae?
- Do the tested substances cause malformations of the cells in the exposed tissues?
- Do the tested substances affect the biochemical parameters of the exposed tissues?
- Do the tested substances affect the physiological parameters of *T. molitor* larvae?
- Do the effects of the extract differ from the effects of pure GAs, and (if yes) what aspects of the toxicity may be caused by solasonine, solamargine or other compounds of the extract?

To answer these questions, we conducted some observational studies and tests of various levels of biological organization. This study included an analysis of the general toxic activity of the *S. nigrum* extract given in the food on the growth of *T. molitor* larvae. Since we had already observed some ultrastructural changes in response to exposure to *Solanaceae* plant extracts [2,29], we decided to test the ultrastructure of the midgut and fat body, which are important tissues for the ingestion and distribution of toxic agents within insect bodies. The midgut was directly exposed to the agents present in the ingested feed. To complement the changes that were observed with electron microscopy, biochemical assays of parameters, such as the content of lipids, glycogen, and proteins in the fat body were conducted. Next, further studies included the analysis of the influence of the extract and pure glycoalkaloids on the visceral muscles and myocardium contractile activity under in vitro conditions, to check their utility as possible factors affecting muscle activity. The modulation of muscle contractility of organs, such as the heart, hindgut, or oviduct may result in impaired development, food intake, and reproduction. Hence, the above mentioned parameters may be crucial for better understanding the toxic mode of action of the tested alkaloids, and they may also contribute to the more efficient application of plant derived substances in plant protection. Consequently, this may lead to the decreased use of both synthetic and natural substances in plant protection, with the benefits of limiting treatment of crops and food products and reducing environmental pollution.

## 2. Results

### 2.1. Changes in Body Mass

The average percentage gain in body mass by the control larvae during the experiment was 15.7 ± 0.8% with *n* = 139 (Table 1). None of the larvae died during the experiment. The lowest mean percentage weight gain (13.4 ± 1.61%) was obtained after solasonine application to the diet at a concentration of 7.52 × 10^−6^ M (Table 2), and the highest (19.1 ± 1.28%) after the application of solamargine in the concentration 7.23 × 10^−6^ M.

### 2.2. Effects on Visceral Muscle Contractility In Vitro

#### 2.2.1. Heart Activity

**Figure 1:**
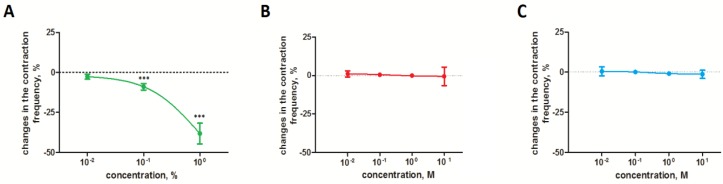
The contraction frequency of *T. molitor* heart after the application of the *S. nigrum* extract (**A**) and pure glycoalkaloids (solamargine (**B**) and solasonine (**C**)). *** Statistical significance at *p* ≤ 0.001, Kruskal-Wallis test with Dunn’s test.

**Figure 2:**
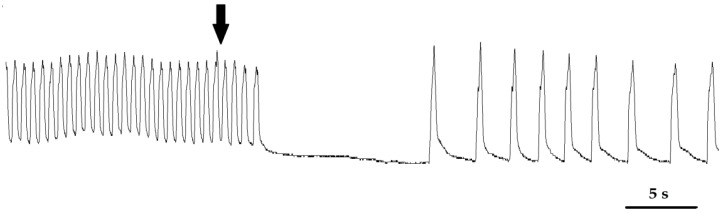
Sample myocardiogram of an adult *T. molitor* beetle. The arrow shows the moment the 1% *S. nigrum* extract was applied.

The extract that was applied to the heart caused a negative chronotropic effect, the strength of which increased with increasing concentration (Figure 1). The strongest effect was observed after application of the 0.1% and 1% extracts. In these cases, an average percentage regarding the lowering of the heart rate of −8.3 ± 1.61% and −40.4 ± 4.58%, respectively, was observed. 1% solution also caused the reversible inhibition of heart activity (Figure 2). None of the tested GAs caused a significant effect on heart activity.

#### 2.2.2. Oviduct Contractility

**Figure 3:**
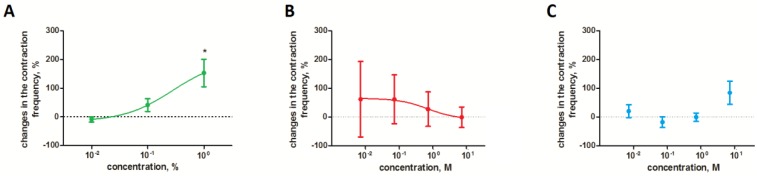
Effect of the *S. nigrum* extract (**A**) and pure glycoalkaloids (solamargine (**B**) and solasonine (**C**)) on contractile activity of *T. molitor* oviduct. * Statistical significance at *p* ≤ 0.05, Kruskal-Wallis test with Dunn’s test.

In contrast to the heart, the 1% extract applied to the oviduct increased the contraction frequency of this organ by an average of 152.7 ± 47.79%. The observed effect was dose dependent, and the intensity of the response increased with an increasing extract concentration (Figure 3A). In the case of solamargine, we also observed a slight increase in the oviduct contraction frequency after application of the glycoalkaloid (Figure 3B). However, the relationship between the strength of the observed effect and concentration was opposite to that caused by the extract.

#### 2.2.3. Hindgut Contractility

**Figure 4:**
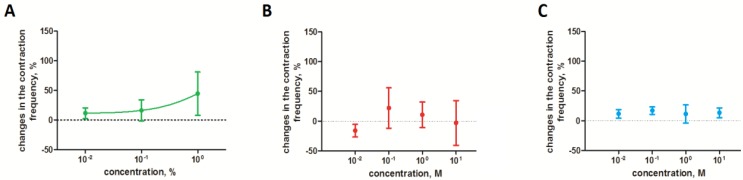
Effect of the *S. nigrum* extract (**A**) and pure glycoalkaloids (solamargine (**B**) and solasonine (**C**)) on contractile activity of *T. molitor* hindgut. ANOVA with Tukey’s test or Kruskal-Wallis test with Dunn’s test.

Similar to the oviduct, the *S. nigrum* extract increased the frequency of the hindgut contraction; nevertheless, the observed effect was definitely slighter (Figure 4A). None of the pure alkaloids that were applied on the isolated hindgut caused a significant effect (Figure 4B,C).

### 2.3. The Influence on the Fat Body and the Midgut Ultrastructure

#### 2.3.1. Midgut

**Figure 5:**
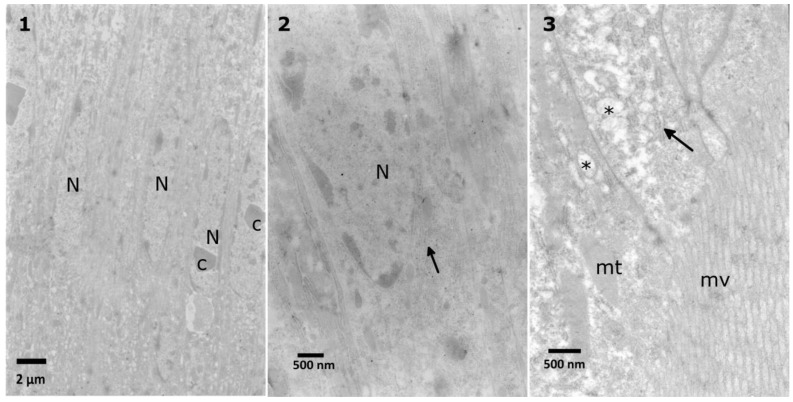
Control cells of the *T. molitor* larvae midgut. The nuclei (N) with protein crystals (c) (**1**) are surrounded by cytoplasm rich in endoplasmic reticulum (arrows) (**2**). The apical part of the cells (**3**) contains many mitochondria (mt), pinocytic vesicles (asterisks) and microvilli (mv).

The columnar midgut cells of *T. molitor* (Figure 5) are characterized by nuclei surrounded by cytoplasm containing rough endoplasmic reticulum (RER), Golgi bodies, and elongated shaped mitochondria. Each nucleus usually contains a single or double protein crystal (Figure 5, No. 1) [30]. The apical part of the cell includes long microvilli that take part in the absorption of digested food. When considering the high metabolic rate and function of midgut cells, the apical zone contains many mitochondria, smooth endoplasmic reticulum (SER), pinocytotic vesicles, and lysosomes. Special attention was paid to the abovementioned zone of the cells because it is the first zone to have contact with digested compounds (in this study, potentially the administered glycoalkaloids).

**Figure 6:**
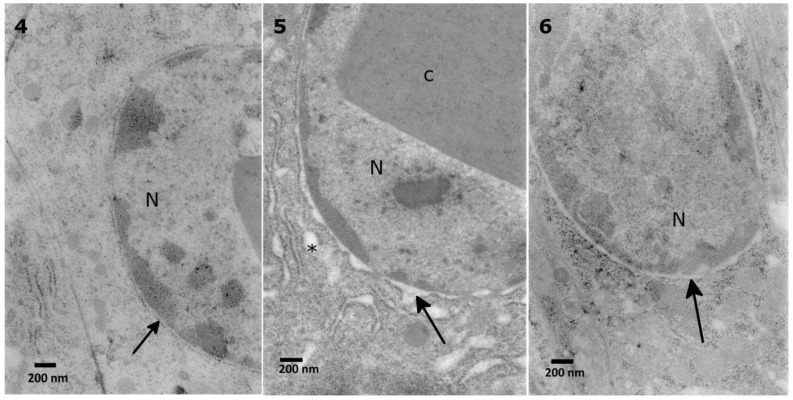
Nuclear (N) membranes in the *T. molitor* midgut cells. The membranes in the control cell (**4**) adhere to each other (**4**, arrow). The membranes are disturbed after application of 0.1% (**5**, arrow) and 1% *S. nigrum* extract (**6**, arrow). The disturbance is also observed in the endoplasmic reticulum (**5**, asterisks).

**Figure 7:**
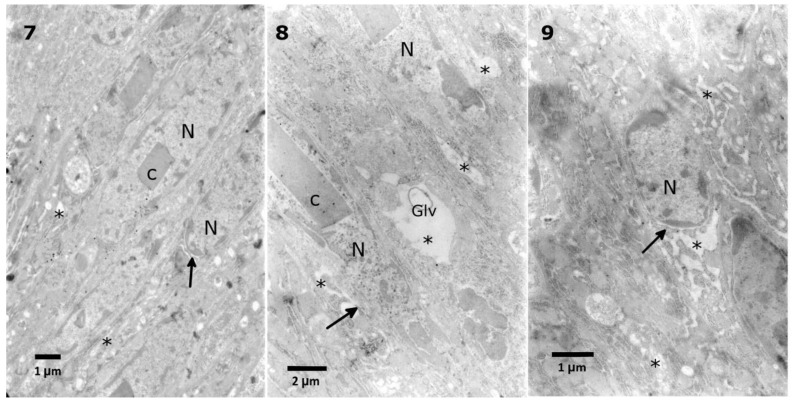
Malformations of nuclear membranes (**7**, arrow) and vacuolization in the cytoplasm (**7**, asterisks) after application of a 1% *S. nigrum* extract in the *T. molitor* midgut cells. The 10% extract caused vacuolization in the cytoplasm (**8**, **9** asterisks) with the appearance of glycogen vacuoles (**8**, Glv) and disturbance of nuclear membranes (**8**, **9**, arrows).

The lowest extract concentration did not cause any evident changes in the ultrastructure in comparison to the control observations. First, mild effects, such as the disruption of the nuclear membranes and swollen perinuclear space, were observed after application of the 0.1% extract (Figure 6 No. 5). After the application of 1% extract, the same effect was observed, with additional changes in the density of the cytoplasm (Figure 7, No. 7). The strongest effects were observed after the application of the 10% extract (Figure 7, No. 8, 9). The nuclear membranes were separated in the basal part of the cells (Figure 7, No. 7, 9). Additionally, a decrease in the cytoplasm density was observed, especially around the nuclei, with the presence of single-membranous structures, most likely glycogen vacuoles (Figure 7, No. 8, Glv), which may be associated with glycogen redeployment [31]. When solasonine or solamargine were added to the diet, no significant changes were observed in the ultrastructure of midgut cells at any tested concentration.

**Figure 8:**
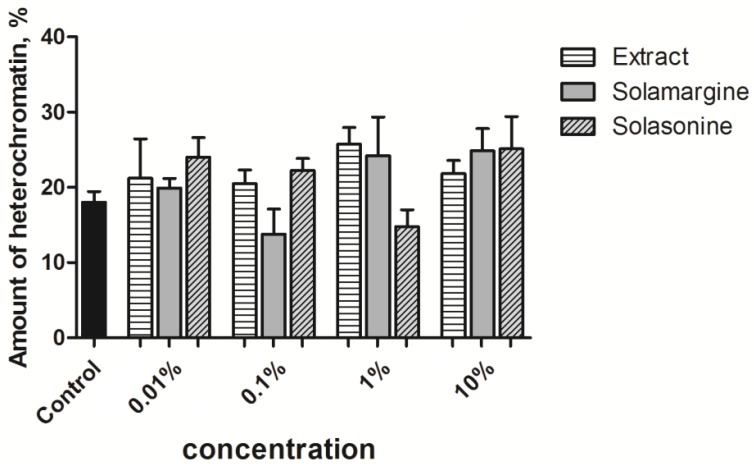
Heterochromatin ratio in the columnar midgut nuclei exposed to various concentrations of tested substances. Solasonine and solamargine were used in concentrations equivalent to their concentrations in the extract (Table 2). Kruskal-Wallis test with the Dunn’s test.

The tested substances did not significantly alter the amount of electron dense chromatin within the nuclei (Figure 8). The correlations between the substance concentration and heterochromatin ratio for the extract, solamargine, and solasonine were 0.06, 0.60, and 0.43, respectively. Only the relationship between the solamargine concentration and heterochromatin ratio could be regarded as a moderate positive correlation.

#### 2.3.2. Fat Body

**Figure 9:**
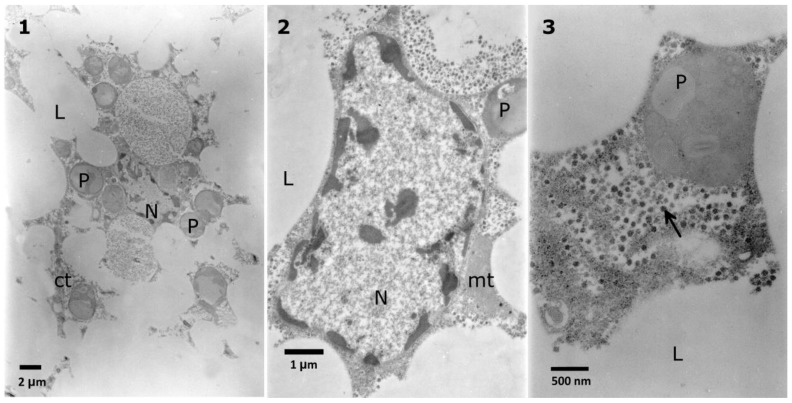
Control cells of the *T. molitor* larval fat body. The cells contain a nucleus (N) (**1,2**), lipid droplets (L), stored proteins (P), mitochondria (mt) (2), and cytoplasm (ct) with glycogen granules (**3**, arrow).

**Figure 10:**
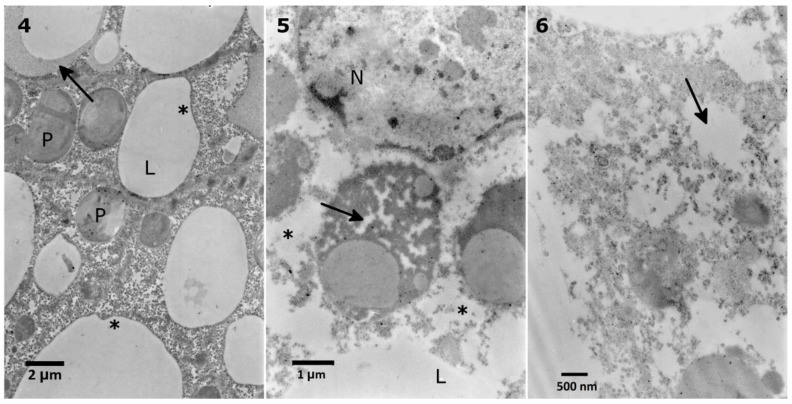
Fat body cells of the *T. molitor* larvae treated with 0.1% *S. nigrum* extract showed changes in the lipid droplet homogeneity (**4**, arrow) with changes in shape regularity (**4**, asterisks). The 1% extract caused disintegration of the stored proteins (**5**, arrow), and vacuolization of the cytoplasm (**5**, **6**, arrows).

**Figure 11:**
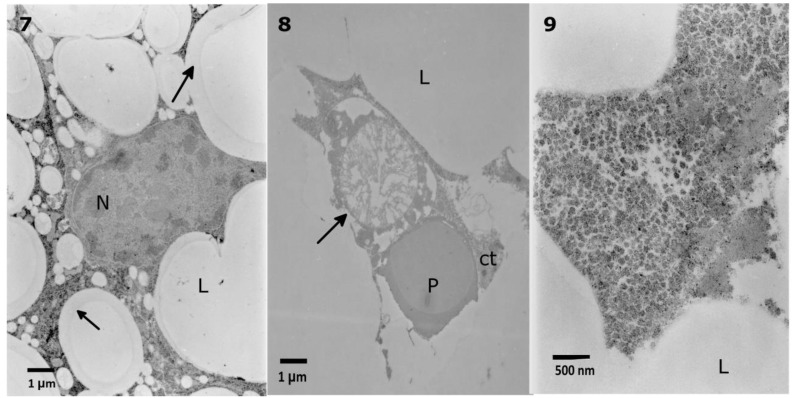
The nuclei (N) of the fat body cells after application of the 10% *S. nigrum* extract to the *T. molitor* larvae showed increased density. In the lipid droplets (L), the homogeneity decreased (**7**, arrow), with the appearance of other nonhomogeneous structures (**8**, arrow). The cytoplasm density increased (**9**).

The observed trophocytes (Figure 9, No. 1) possessed regularly shaped lipid droplets of various sizes, stored proteins, and cytoplasm filled with glycogen granules. The control cells had regular nuclei, with heterochromatin patches being located in the center and in the vicinity of the nuclear envelope (Figure 9, No. 2). The glycogen granules in the cytoplasm of the control cells appeared to be uniformly distributed. After the application of the extract at a concentration of 0.1%, some of the lipid droplets lost their homogeneity and regularity in shape (Figure 10, No. 4). Increasing the extract concentration to 1% caused a disruption of stored proteins and a decrease in the cytoplasm density (Figure 10, No. 5, 6). As in the case of the midgut cells, the most visible effects were observed after the application of the 10% extract, where the appearance of disrupted proteins and lipids were the most prominent changes. In many cases, nuclei with very dense nucleoplasm were observed (Figure 11, No. 7).

**Figure 12:**
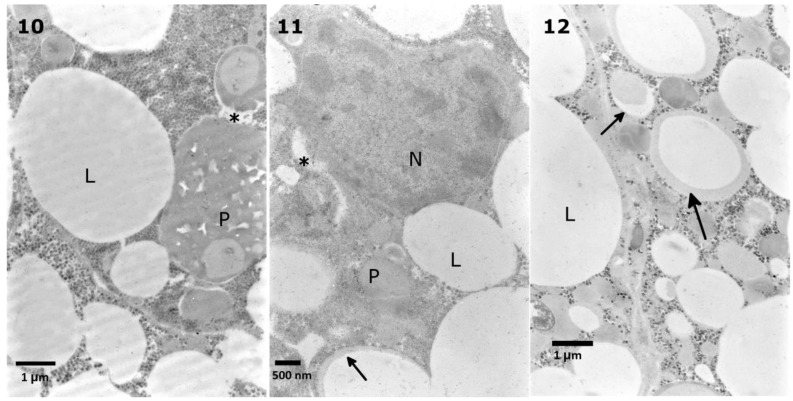
The fat body cells of the *T. molitor* larvae showed changes in the stored protein homogeneity (**10**, P) after application of solamargine at a concentration of 7.23 × 10^−7^ M and in the lipid droplet homogeneity (**11**, arrow) after the application of the 7.23 × 10^−5^ M concentration. Similar changes in the lipid droplets homogeneity were observed after the application of a concentration of 7.23 × 10^−4^ M. Slight changes in the cytoplasm density were observed after the application of solamargine at concentrations of 7.23 × 10^−7^ M and 7.23 × 10^−5^ M (**11**, **12**, asterisks).

The application of solamargine at a concentration of 7.23 × 10^−7^ M caused an increase in the cytoplasm density, but with areas of vacuolization and a change in the homogeneity of the stored proteins (Figure 12, No. 10). An increase in the applied concentration to 7.23 × 10^−5^ M caused slight changes in the lipid droplets homogeneity and an increase in the cytoplasm density. Some observed nuclei showed the increase of the nucleoplasm density (Figure 12, No. 11). The strongest concentration of solamargine, 7.23 × 10^−4^ M, also caused changes in the lipid droplet homogeneity (Figure 12, No. 12).

**Figure 13:**
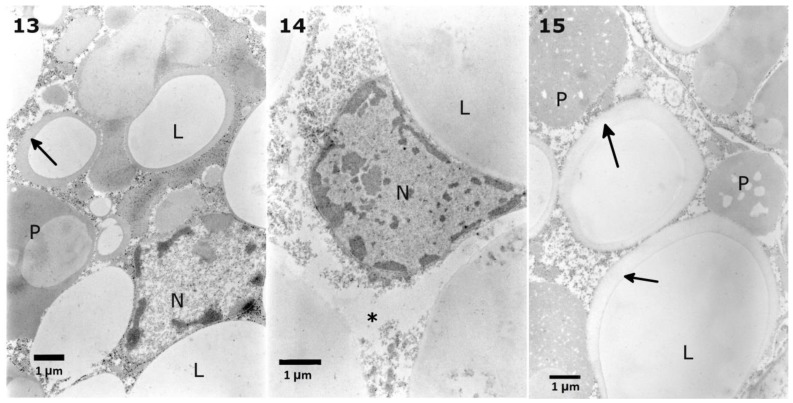
Solasonine given in the diet of the *T. molitor* larvae caused changes in the lipid droplet homogeneity after the application of concentrations of 7.52 × 10^−7^ M (**13**, arrow) and 7.52 × 10^−4^ M (**15**, arrows). The concentration 7.52 × 10^−5^ M caused a decrease in the cytoplasmic density (**14**, asterisk). After the application of a concentration of 7.52 × 10^−4^ M, initial disintegration of the stored proteins was observed (**15**, P).

Solasonine, at a concentration of 7.52 × 10^−7^ M, caused changes in the lipid droplet homogeneity (Figure 13, No. 13). Similar observations were noted after application of the 7.52 × 10^−4^ M concentration (Figure 13, No. 15). This concentration also caused the disintegration of the stored proteins. The decrease in the cytoplasm density with areas of vacuolization was observed after the application of solasonine at a concentration of 7.52 × 10^−5^ M (Figure 13, No. 14).

**Figure 14:**
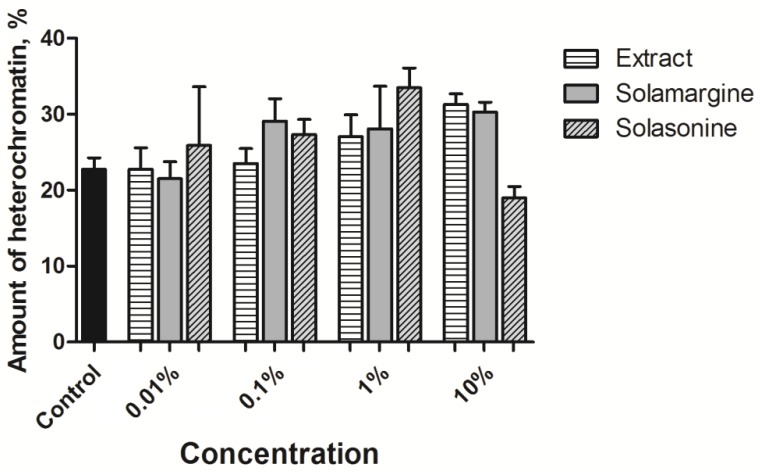
Heterochromatin ratio in the nuclei of the fat body cells exposed to various concentrations of tested substances. Solasonine and solamargine were used in concentrations equivalent to their concentrations in the extract (Table 2). Kruskal-Wallis test with Dunn’s test.

In the case of the extract and solamargine, we noted a statistically non-significant, but evident increase in the heterochromatin ratio within the nuclei that is positively correlated with the increasing concentration of the tested substance (Figure 14). In the case of solasonine, the tendency was clear for the three lower concentrations but the low ratio that was calculated for the highest one weakened the overall trend. Additionally, the values of the correlation coefficients indicated a strong positive correlation between the extract and solamargine concentrations, and the heterochromatin ratio (0.75 and 0.78, respectively). The correlation coefficient for solasonine showed a very strong correlation ranging from 0.01% to 1%, but the highest concentration drastically decreased the coefficient to a negative value (−0.77).

### 2.4. Biochemical Assays of the Fat Body Cells

#### 2.4.1. Glycogen

**Figure 15:**
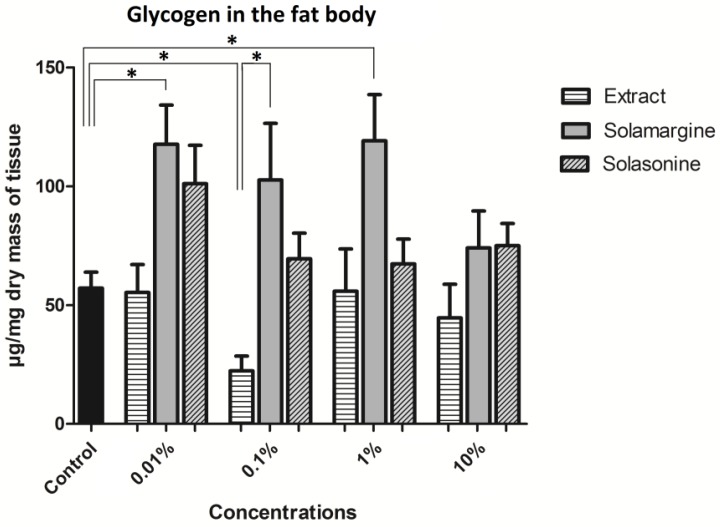
The level of glycogen in the fat body of the *T. molitor* after the application of extract from *S. nigrum* and solasonine and solamargine in their molar concentrations equal to their concentrations in the applied extract concentrations. * Statistical significance at *p* ≤ 0.05; Kruskal-Wallis test with Dunn’s test, *n* ≥ 12.

Both the extract and solamargine caused changes in the glycogen level of the fat body as compared to the control (Figure 15). No significant changes were observed after solasonine application to the diet of the larvae. The average amount of glycogen in the fat body that was isolated from the control insects was 57.1 ± 6.73 μg/mg of dry mass of the tissue. The extract caused a significant decrease in the glycogen content in the fat body to 22.3 ± 6.27 μg/mg after the application of the 0.1% concentration. Solamargine significantly increased the amount of the glycogen in the fat body at concentrations ranging from 7.23 × 10^−7^ M to 117.7 ± 16.47 μg/mg, and 7.23 × 10^−5^ M to 119.2 ± 19.41 μg/mg as compared to the control. The application of solasonine increased the glycogen content in the fat body. The strongest effect was observed at the lowest tested concentration, which caused an almost two-fold increase in the glycogen content nevertheless, the change was statistically non-significant. Between the effects that are caused by the extract and the testes pure GAs, in general, the glycogen level was higher after the application of either of the GAs than that of the extract application. A significant difference was achieved at a concentration of 0.1%, where the extract decreased and solamargine and solasonine increased the level of glycogen in the fat body (Figure 15).

#### 2.4.2. Lipids

**Figure 16:**
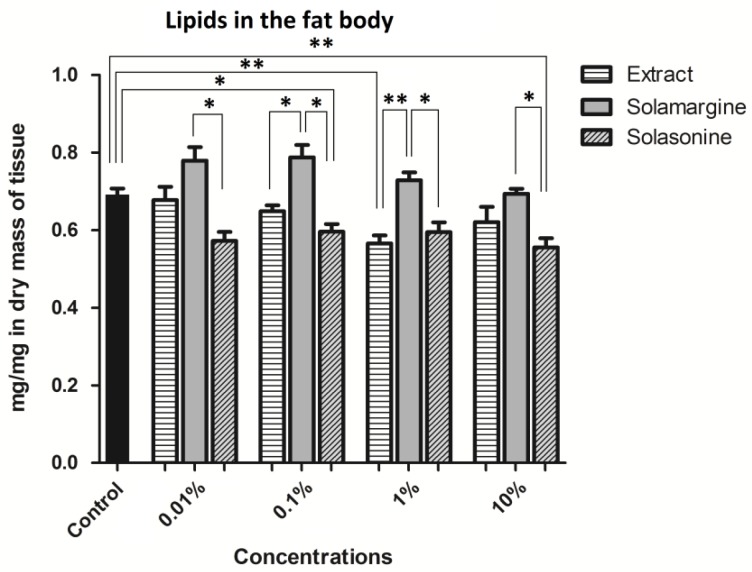
The content of lipids in the fat body of the *T. molitor* larvae after application of the extract from *S. nigrum* and solasonine and solamargine in their molar concentrations equal to their concentrations in the applied extract concentrations. ** Statistical significance at *p* ≤ 0.01, * *p* ≤ 0.05, one-way ANOVA, Tukey’s test, *n* ≥ 9.

Lipids are the main ingredient of the fat body, representing more than 50% of the dry weight of the tissue [32]. The measured mass of the lipids in this study included not only the components of the lipid storing vacuoles, but also, for example, membrane lipids. However, the inclusion of the other lipids in the obtained results was negligible. In the control, the average lipid content in the dry mass of the fat body was 0.69 ± 0.02 mg/mg of dry tissue (Figure 16). At a concentration of 1%, the extract lowered the lipid content in the fat body after application to 0.57 ± 0.02 mg/mg. Solasonine also significantly decreased the lipid content in the fat body to 0.6 ± 0.02 mg/mg and 0.56 ± 0.02 mg/mg in comparison to that of the control at concentrations ranging from 7.52 × 10^−6^ M to 7.52 × 10^−4^ M, respectively. Differences between the extract and solamargine were observed for concentrations 0.1% (*p* ≤ 0.05) and 1% (*p* ≤ 0.01), and the lipid amount in the fat body was lower after extract application than after solamargine application. At all of the tested concentrations, significant differences were observed between solasonine and solamargine, where a higher content of lipids was present after solamargine application and lower after solasonine application.

#### 2.4.3. Proteins

**Figure 17:**
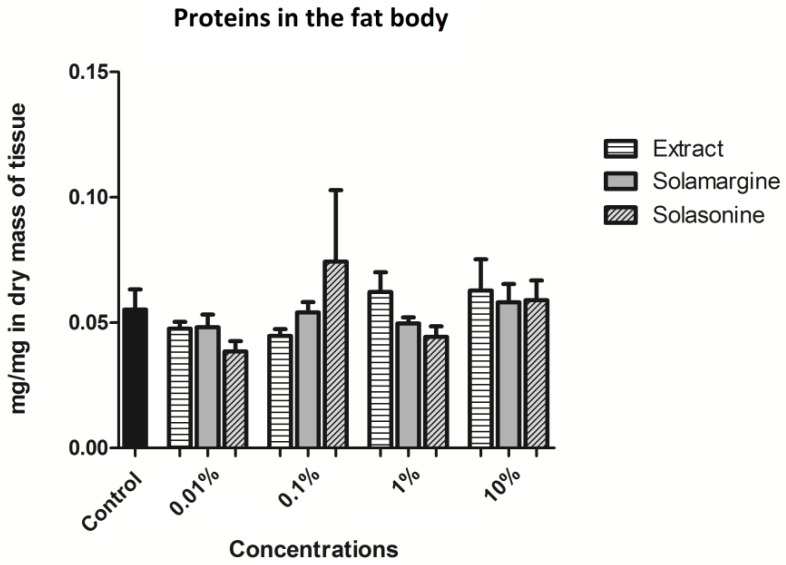
The content of the soluble proteins in the fat body of the *T. molitor* larvae after application of the extract from *S. nigrum* and solasonine and solamargine in their molar concentrations equal to their concentrations in the applied extract concentrations. Kruskal-Wallis test with Dunn’s test, *n* ≥ 13.

The content of the soluble proteins in the fat body did not show significant changes after application of the extract or GAs. In the control, the average protein content was 0.06 ± 0.008 mg per mg of dry mass of the tissue (Figure 17).

## 3. Discussion

In the present study, an expanded description of the effects that are caused by the extract of *S. nigrum* and pure GAs on *T. molitor* physiology was conducted. The tested substances do not appear to have acute toxic effects on the *T. molitor* larvae. In addition, the results differed between the extracts and pure GAs. The results showed that the exposure of the larvae to the tested substances caused slight changes in the body mass, ultrastructure of the midgut and fat body, and biochemical parameters of the fat body. However, it must be noted that these results were obtained over the relatively short observation periods that were used for the experiments. Hence, the observed effects often showed a trend, but the changes were not always statistically significant.

At the subcellular level, one can observe the very early effects, which appeared only in some cells. Therefore, the observed malfunctions and malformations can be used as bioindicators of stress caused by toxic substances. Perhaps, extended exposure or increased dosage would have given more significant effects. However, the aim of the study was to examine the direct, immediate effects and the mechanisms of the toxicity of *S. nigrum* extract and pure GAs. Furthermore, sublethal effects are often very important, they can limit crop or stored food loss [2] or affect the cost-to-benefit ratio similar to that obtained with synthetic insecticides [33].

The weight of the larvae did not change significantly but some tendencies were observed. It appears that the plant-derived substances may cause bimodal effects. For example, exposure to the low concentrations of solasonine slightly decreased the larval gain in body mass, while higher concentrations did not cause such an effect. On the other hand, only the highest concentration of the extract decreased the body mass, and the lower concentrations increased the body mass. Alkaloids in high concentrations may deter herbivores from feeding, as usually they are present in unripe fruit. Glycoalkaloids, such as solamargine and solasonine, have been tested by Weissenberg et al. [8] on *Tribolium castaneum*, belonging to the same family (Tenebrionidae) as *T. molitor*. The results showed that these compounds acted as growth inhibitors in larvae, but the experiment lasted 15 days. This finding also proves that GAs may be useful in plant protection in sublethal doses, thus limiting pest feeding. The results indicate that the plant must produce other substances that are responsible for the limitation of feeding and the growth of herbivores.

The effects on muscles differed between the tested substances. The application of the extract altered the contraction of heart, oviduct, and hindgut musculature, whereas the effects of solasonine and solamargine were much weaker, with solasonine decreasing the oviduct activity. These data suggest that neither solasonine or solamargine was responsible for the observed effects after application of the extract and indicate that plant extracts must contain other active substances in addition to the main GAs, perhaps alkaloids present in a lower concentrations, which affect muscle physiology. Both the glycoalkaloids may influence each other or interact with other ingredients of the extract. A stronger reaction of the insects to extracts than to single compounds has been described previously [12,34]. Moreover, the opposite effect was observed after the application of the extract to the heart, where the contraction frequency decreased, as compared to that on the oviduct, where the frequency increased. This finding suggests that the extract has a different mode of action on the two muscle tissues, and its components may interact with different receptors. The obtained results are similar to those that were obtained by Ventrella et al. [34], where black nightshade extract was used on the heart of *Zophobas atratus* (Tenebrionidae), and at a concentration of 0.5 mM, it caused a reversible negative chronotropic effect.

The microscopic observations revealed the differences between the effects caused in both of the tissues. Perhaps the differences were caused by a longer time of exposure of the fat body than of the midgut. Columnar cells transport ingested substances to other tissues, and trophocytes store substances and play crucial roles in detoxification. Therefore, these cells can reveal more drastic effects. A similar phenomenon was observed in the case of exposure to other toxic substances, such as boric acid [35] and tomato or potato leaf extract [36]. Although the intensity of the ultrastructural malformations differed between the tested organs, species, and substances, some of those effects were similar: swollen nuclear envelopes and endoplasmic reticulum, cytoplasmic vacuolization, or swollen mitochondria have been reported for many toxic substances, including synthetic pesticides [16,37] or plant-derived substances [15,29,38,39]. These ultrastructural changes may be due to the increased production of reactive oxygen species, which has been reported for various toxic substances [35,40,41,42]. It is noteworthy that the biochemical parameters of the fat body depend on the biochemical parameters of the hemolymph, which transfers compounds to and from the fat body. Therefore, in the near future, we plan to further observe of the hemolymph biochemical parameters, such as sugar and lipid levels, and to correlate the obtained results with those of the fat body biochemistry and ultrastructure. To compare the effects that are caused by the *S. nigrum* extract and its pure GAs, both of the substances were given to the larvae and changes in the ultrastructure of the midgut and fat body cells were observed. While the extract caused a disruption of the nuclear membranes and cytoplasm vacuolization in the midgut, solasonine, and solamargine did not show any visible effects on these cells. This suggests that other glycoalkaloids present in the extract may be responsible for the observed effect or that solasonine and solamargine act synergistically in the extract or that the other compounds found in the extract play crucial roles in the process of membrane lysis by GAs.

Ultrastructural studies showed changes in the chromatin condensation that could lead to the altered expression of genes. The results suggest that solamargine is mostly responsible for chromatin condensation. For both tested concentrations of solamargine, the correlations with the effects on chromatin condensation were the strongest observed in this study. On the other hand, bimodal effects were observed in the case of solasonine in the fat body cells. Therefore, the correlation coefficient cannot be treated here for the whole range, as it measures linear relationships. The condensation of chromatin was also reported for cancer cells that were exposed to solamargine [43]. Solasonine and solamargine are both glycosides of solasodine (aglycone, the true alkaloid). They are characterized by sugar moieties and aglycone moieties. The electrophilic behavior of these compounds is regulated by the polarity of the sugar moieties and by the presence of oxygen and nitrogen ion pairs in the aglycone moiety, which does not contain aromatic rings. These features are responsible for GA-induced membrane disruption and interactions with nucleic acids, which result in DNA malformations [44,45]. Again, the weak effects that were observed in this study probably reflect the relatively low doses of applied substances that affected the nuclei in both the tested tissues. However, the teratogenic activity of GAs has been proven [46,47,48] and it may be due to the disruptive effect of GAs on membranes and nucleic acids.

The results of the biochemical studies showed that the level of glycogen decreased significantly after the application of the extract at only one concentration 0.1% (Figure 15). Glycogen is the first source of glucose during periods of starvation or detoxification; hence, one can suppose that the extract might have had a significant effect on the larvae. However, the weight gain of the larvae treated with this extract concentration was the highest among the used concentrations, which did not confirm the theory of starvation. Rather, glycogen, as an energy source, might have been used for detoxification and the vitality of the insects was not decreased. A slight decrease in the glycogen content of the fat body was also observed after application of the extract at a concentration of 10%, and the percentage of the larval weight gained seemed to decline. Perhaps a longer exposure would demonstrate whether this effect could be intensified. Such an effect suggests that *S. nigrum* produces substances that can be used as deterrents. Satake et al. [49] observed a decrease in the fat body glycogen content after starvation of the larvae of *Bombyx mori*, and that can be an early bioindicator of the inhibition of food intake. Another place, where carbohydrates are stored by insects and are transported to or from the fat body, is the hemolymph, which was not studied in our research. Previous studies claimed that the hemolymph glucose level can be an indicator of fat body carbohydrate metabolism [50]. The correlation between these factors results from the inhibition of glycolysis in the fat body by the key enzyme regulating this process present in the hemolymph—fructose-2,6-biphosphate. Solamargine caused an increase in the glycogen content, as compared to that of the control. The difference was significant after application of the concentrations of 7.23 × 10^−7^ M and 7.23 × 10^−5^ M. The increase in the glycogen amount may be connected with the increase in the food intake by the larvae, which corresponds with the percentage body mass gain after solamargine application. The results showed the highest body mass gain in the case of solamargine. However, in general, the content of glycogen in the fat body was higher after the application of both GAs than after the extract application. A significant difference was obtained between the extract 0.1% concentration and the equivalent concentration of solamargine, which suggests different modes of action in the extract.

*S. nigrum* extract significantly decreased the lipid content in the fat body (Figure 16) after application of the 1% concentration. This result does not correspond with the gain in body mass, which increased when compared to that of the control. One supposes that the reason for this discrepancy may be, again, not the observed level of lipids in the hemolymph. Lipids from the fat body could have been moved to the hemolymph, which may have caused the decrease in the lipid levels of the fat body, without changing the body mass of the insects. Lipid mobilization is activated during starvation stress [50], which supports the theory that food intake is inhibited by alkaloids. Significant differences were present not only between the level of lipids in the control and extract treatment groups, but also between the extract and solamargine treatment groups. These data show, similar to the case of glycogen, that in the extract, other substances can be present that modulate its mode of action and play a crucial role in the toxic action of the major GAs. Significant differences in the lipid content were obtained between both the GAs at all the tested concentrations. These results show different influences of both the GAs on the lipid metabolism in the fat body.

No significant changes in the protein content were observed after the application of the tested substances. Only slight changes in the amount of protein were present when compared to that of the control. However, the quantity of protein was measured, but their profiles were not assessed. We do not know if some proteins are specifically produced, which would not necessarily influence the general protein content of the fat body, but might greatly alter its metabolism. This is a very interesting question that we will address in future studies. Furthermore, the observed changes in the chromatin density of the fat body, as well as its other ultrastructural alterations, suggest that the metabolism of the fat body may change dramatically during exposure to the GAs and their detoxification.

The difference between the effects that were observed with the transmission electron microscope and the biochemical analyses is also of interest. The altered homogeneity of the lipid droplets, cytoplasm density, and therefore glycogen content, as well as the stored protein disintegration observed by microscopy, did not strongly correspond with the results of the biochemical analyses. The main reason for this difference is that the biochemical analyses were conducted on dry tissue. In addition, it is very likely that the ultrastructural changes occurred before they could be analyzed biochemically and before significant lethality appeared. These results suggest that the use of transmission electron microscopy as a tool for early changes in detection, especially when employing short tests periods is highly justifiable. Furthermore, this implies that ultrastructural malformations may be used as environmental and functional bioindicators of exposure to the low concentrations of stressors, or they may represent the early stage of exposure.

## 4. Conclusions

The results indicate that both the extract and pure GAs have a wide range of sublethal effects. Although the effects do not cause mortality in the larvae, they may disturb the insects’ development and metabolism at various levels. The observed modulation of muscle contractility of such organs as the heart, hindgut, or oviduct may result in impaired development, food intake, and reproduction. Hence, the above mentioned parameters may be crucial for better understanding the mode of toxicity of the tested alkaloids. Furthermore, these studies may also contribute to the more efficient application of plant-derived substances for plant protection. Consequently, this may lead to a decreased usage of both synthetic and natural substances in plant protection, which may limit the pollution of the environment, crops, and food products. It is noteworthy that some effects were observed very early, after exposure. Therefore, they may be useful as bioindicators of stress. Moreover, they may be used to limit the pest population by decreasing the vitality of insects. Consequently, they may decrease herbivory. Interestingly, the effects of the extract and pure alkaloids differ from each other. These data suggest that substances produced by the plant may act additively or synergistically. Hence, the effect of the extract may be more intense than that of pure glycoalkaloids. Furthermore, this suggests that plant extracts not only are an interesting source of new insecticides, but also may be used as relatively inexpensive tools in plant protection, especially when integrated pest management strategies are applied.

## 5. Materials and Methods

### 5.1. Insects

*Tenebrio molitor* larvae and adult beetles were obtained from the breeding culture at the Department of Animal Physiology and Development (AMU) under laboratory conditions; the experimental specimen were maintained at 26 °C and a 60% relative humidity in a 12 h light to 12 h dark photoperiod. For the experiments, four-week-old adults and larvae after molting with weights of 120–140 mg were used. Determining insect weight allowed us to choose the larvae with the same metabolic rate and to control for weight gain.

### 5.2. Extraction and Analyses

Extracts were obtained from *S. nigrum* unripe berries. The voucher specimens were deposited at the Herbarium Lucanum (HLUC, Potenza, Italy), with the ID Code: 2320. The extraction method was previously described by Cataldi et al. [51] and Adamski et al. [41]. The berry samples were lyophilized and ground to a fine powder using a laboratory mill. The samples (1.5 g) were placed in 20 mL of 1% acetic acid aqueous solution. The suspension was stirred for 2 h and then centrifuged at 6000 rpm for 30 min. The obtained pellet was suspended in 5 mL of 1% acetic acid, shaken, and centrifuged. Two supernatants were subsequently mixed together. The extract was filtered through a single-use 0.22 μm nylon filter (Whatman, Maidstone, UK) and then injected into the LC/MS system. The chemical analysis was conducted at the Department of Sciences, University of Basilicata by Prof. Sabino Bufo’s team.

The extracts at concentrations of 0.01, 0.1, 1, and 10% were diluted in physiological saline A (274 mM NaCl, 19 mM KCl, 9 mM CaCl_2_, 5 mM glucose, and 5 mM HEPES, pH 7.0) for in vitro experiments or in saline B (274 mM NaCl, 19 mM KCl, 9 mM CaCl_2_) for in vivo experiments. Pure solasonine and solamargine were purchased from Glycomix (Glycomix Ltd, Compton, Berkshire, UK). The standard glycoalkaloids were diluted in the physiological saline A or B for in vitro and in vivo experiments, respectively. The concentrations of both glycoalkaloids were calculated as an equivalent of their quantity in the tested extract (Table 2).

### 5.3. Exposure of the Larvae to the Tested Substances

#### 5.3.1. In Vitro Heart Bioassay

The in vitro effects of the tested extract and GAs on *T. molitor* heart were measured with a microdensitometric technique [52]. Anesthetized four-week-old adult insects were decapitated and their legs and wings were cut off. The ventral cuticle was removed with narrow stripes left the on sides. The visceral organs were carefully removed to expose the myocardium. In the next step, the semi-isolated hearts were placed in a superfusion chamber with the open-perfusion system being mounted in the microdensitometer MD-100 (Carl Zeiss, Jena, Germany) and were perfused with saline A. The flow rate of saline A was 300 μL/min, wherein the solution was continuously removed from the superfusion chamber by chromatographic paper (Whatman No. 3, Sigma-Aldrich, St. Louis, MO, USA). Ten microliters of the tested substances were applied with a Hamilton syringe through the application port placed 70 mm above the superfusion chamber. After recording the control heart activity for 0.5 min, the tested compounds were applied, and an additional 2 min of heart activity was recorded. The recording of new-isolated heart activity was preceded with 10 min of preincubation with saline A to stabilize the heart rate. The calculation of changes in the heart rate was conducted according to a previously described method [53], as the percentage change between the heart activity recorded before and after application. The obtained data were analyzed with LARWA and ANALIZA software (Both programs have been written in 2004 for our Department, Poznań, Poland). For each concentration, 5–12 larvae were used.

#### 5.3.2. In Vitro Oviduct and Hindgut Bioassay

To analyze the changes in the contractile activity of the oviduct and hindgut treated with the tested compounds, a video microscopy technique was used [54,55]. Four-week-old insects of *T. molitor* beetle were anesthetized for 8 min. Next, the insects were decapitated and the legs and wings were removed. The isolation of the oviduct with ovaries was conducted after removing the dorsal cuticle. Next, the preparation was carefully cleaned of undesirable tissues, such as the fat body and Malpighian tubules with microsurgical forceps. The oviducts with ovaries were placed on the Sylgard elastomer and attached with Minuten pins. A similar procedure was used for the isolation of the hindgut. The organs were placed in the incubation chamber with a constant flow rate of 300 μL/min of saline A. The chamber was placed on an Olympus SZX12 stereomicroscope that was equipped with a Pixeling 662 camera. Similar to the heart bioassay, the recording was preceded by 10 min of preincubation. Each recording lasted 2 min; after 30 s of control recording, 10 μL of the tested substances was applied through the port with the Hamilton syringe. The obtained data were analyzed with the AnTracker (PreOptic, Warsaw, Poland) software. The changes in the oviduct and hindgut contraction frequency were calculated as the percentage change between the frequencies recorded before and after application of the tested compounds. For each used concentration, 6–13 larvae were used.

#### 5.3.3. Determination of Changes in Larval Weight

The larvae were kept separately in the flasks. The day after collection, insects were fed for three days with a recipe prepared according to David et al. [56] containing 10 μL of the tested substances. The control larvae were fed with the same mixture containing 10 μL of saline B. On the fourth day of the experiment, the larvae were weighed and the samples were collected according to the further description.

The larvae fed for three days with the extract, solasonine, or solamargine, were weighed before and after the experiment. The number of larvae used varied from 32 to 139 individuals per concentration used. The difference between the weights before and after the experiment of larvae that werefed with the tested substances was compared to that of the control larvae.

The change in body mass was calculated according to the following Equation:

(1) $$\Delta \;=\;\left(\frac{b\times 100}{a}\right)-100$$

where ‘*a*’ is the mass of larva before and ‘*b*’ is the mass after the experiment.

#### 5.3.4. Transmission Electron Microscopy

The larvae that were fed with the extract or pure alkaloids were chosen randomly (three per concentration) and anesthetized with carbon dioxide. Then, they were dissected and the samples of the fat body and midgut were isolated, washed in physiological saline B, and cleaned of other structures, such as the Malpighian tubules and tracheoles. The preparation and fixation of the samples were carried out according to the methods that were described by Adamski et al. [14], as follows: the samples were placed in 2% glutaraldehyde in 0.175 M cacodylate buffer for 2 h, postfixed with 1% osmium tetroxide for 2 h, and finally dehydrated and embedded in Spurr resin (Electron Microscopy Sciences, Hatfield, PA, USA). Ultrathin sections were cut with a Leica ultramicrotome and stained with uranyl acetate and lead citrate. Samples were observed under JEOL 1200EX II JEM (JEOL, Tokyo, Japan) transmission electron microscope. We focused on midgut columnar epithelial cells and fat body trophocytes, as the most frequent cells, which are also crucial for the ingestion, storage, detoxification, and regulation of physiological processes.

The heterochromatin ratio (i.e., electron dense surface to whole nucleus surface area) were calculated using the classic stereological method with the computer programme STEPanizer [57]. Digital grids (1024 squares per picture) were plotted on the images and the number of squares over the electron dense and electron lucent chromatin were counted. Then the ratio was calculated. The mean from a minimum of 8 nuclei per concentration for each test variant was calculated and compared. Next, the correlation between the concentration of the tested substances and the heterochromatin ratio was calculated. Values between −0.3 and 0.3 were regarded as having no linear relationship, values between 0.3 < *x* ≤ 0.5 and −0.3 > *x* ≥ −0.5 indicated a weak (positive/negative) relationship, values between 0.5 < *x* ≤ 0.7 and −0.5 > *x* ≥ −0.7 were regarded as having a moderate (positive/negative) relationship, values between 0.7 < *x* ≤ 0.9 and −0.7 > *x* ≥ −0.9 indicated a strong (positive/negative) relationship, and values between 0.9 < *x* ≤ 1 and −0.9 > *x* ≥ −1 indicated a full (positive/negative) relationship.

#### 5.3.5. Biochemical Analysis of the Fat Body

The samples of the fat body (1–3 mg) after isolation were placed in Eppendorf tubes, then dried under vacuum conditions (−0.9 atm) at 60 °C, and weighed. Next, the glycogen, lipid, and protein content in the samples were determined. The amount of analyzed substances was expressed as milligrams of substances per milligram of dry mass of the tissue. The number of individuals used for each concentration for each test was at least nine.

- **Determination of theglycogen content**

Isolation and determination of glycogen, as described previously by Chowański et al. [58], was carried out according to the procedure of van Handel [59] and Dubois et al. [60], respectively, as follows. Next, 500 μL of 30% KOH was added to the samples and incubated for 15 min at 90 °C to lyse the tissues. After lysis, 50 μL of saturated solution of Na_2_SO_4_ and 800 μL of 96% ethanol were added to precipitate the glycogen. Next, the samples were centrifuged at 10,000 rpm for 10 min and the obtained pellet was washed three times with 70% ethanol. After evaporation of residual ethanol at 74 °C, 500 μL of purified water was added. The pellet was shaken for 5 min at 80 °C and then centrifuged for 5 min at 10,000 rpm. The obtained solution was used to determine the glycogen amount. As a standard oyster, glycogen (Sigma-Aldrich, St. Louis, MO, USA) was used.

- **Determination of the lipid content**

The isolation of the fat body lipids was conducted according to the Folch et al. [61] method described previously by Chowański et al. [62]. The tissues were homogenized in 1000 μL of chloroform-methanol mixture (2:1, *v*/*v*) and centrifuged at 10,000 rpm for 10 min. The supernatant was transferred to new Eppendorf tubes and washed three times with 220 μL of 0.29% NaCl. The remaining solution was evaporated at 30 °C under vacuum (−0.9 atm). The pellet was dissolved in 1000 μL of chloroform-methanol mixture and 500 μL of the solution was transferred to the new Eppendorf tubes. After drying under vacuum (30 °C, −0.9 atm), the mass of the residual lipids was measured gravimetrically.

- **Determination of the soluble protein content**

After drying, the samples were homogenized in saline B on ice. Next, they were centrifuged at 10,000 rpm for 5 min. Two microliters of the intranatant was placed on the PTFT membrane, dried, and measured with a Direct Detect^®^ Infrared Spectrometer (Merck Millipore, Burlington, MA, USA).

### 5.4. Statistical analysis

All the data are presented as the mean values ± SEM of *n* number of replicates. The statistical significance of differences between the control and treatment values was determined using appropriate statistical test: one-way ANOVA Tukey’s test, Student’s *t*-test, or, if there was not a normal distribution, the nonparametric Kruskal-Wallis test and Dunn’s Multiple Comparison test. The statistical analyses were conducted using GraphPad Prism 5 software (GraphPad Software Inc, Version 5.01, MacKiev, La Jolla, CA, USA, 1992–2007). Differences were considered to be statistically significant if *p* ≤ 0.05 (*), *p* ≤ 0.01 (**), or *p* ≤ 0.001 (***)

## Figures and Tables

**Table 1:** The percentage gain in body mass by *T. molitor* larvae after application of the extract, solamargine, solasonine and saline B (control) into to the diet. The data are shown as the mean ± SEM. ANOVA, Tukey’s test.

	Concentration	0.01%	0.1%	1%	10%	Control	
Gain inBody Mass (%)							
Extract		18.6 ± 1.28	18.7 ± 1.42	17.5 ± 1.33	15.2 ± 1.5	15.7	± 0.75
Solamargine		18.2 ± 1.31	19.1 ± 1.28	17.0 ± 1.05	16.8 ± 1.34		
Solasonine		13.9 ± 1.38	13.4 ± 1.61	16.5 ± 1.63	16.7 ± 1.42		

**Table 2:** Calculation of the glycoalkaloid concentrations in the extract.

*S. nigrum* Extract Concentration (%)	Solamargine (M)	Solasonine (M)
0.01%	7.23 × 10^−7^	7.52 × 10^−7^
0.1%	7.23 × 10^−6^	7.52 × 10^−6^
1%	7.23 × 10^−5^	7.52 × 10^−5^
10%	7.23 × 10^−4^	7.52 × 10^−4^
